# Geroscience in the Age of COVID-19

**DOI:** 10.14336/AD.2020.0629

**Published:** 2020-07-23

**Authors:** Nir Barzilai, James C Appleby, Steven N Austad, Ana Maria Cuervo, Matt Kaeberlein, Christian Gonzalez-Billault, Stephanie Lederman, Ilia Stambler, Felipe Sierra

**Affiliations:** ^1^Institute for Aging Research, Albert Einstein College of Medicine, New York, NY, USA; ^2^Gerontological Society of America, USA; ^3^Department of Biology, University of Alabama at Birmingham, USA; ^4^Department of Pathology, University of Washington, USA; ^5^Geroscience Center for Brain Health and Metabolism (GERO), Chile; ^6^The Buck Institute for Research on Aging, USA; ^7^American Federation for Aging Research, USA; ^8^Vetek (Seniority) Association - the Movement for Longevity and Quality of Life, Israel; ^9^National Institute on Aging, NIH, USA

**Keywords:** geroscience, COVID-19, immunosenescence, geroprotectors, aging biomarkers

## Abstract

The data on COVID-19 is clear on at least one point: Older adults are most vulnerable to hospitalization, disability and death following infection with the novel coronavirus. Therefore, therapeutically addressing degenerative aging processes as the main risk factors appears promising for tackling the present crisis and is expected to be relevant when tackling future infections, epidemics and pandemics. Therefore, utilizing a geroscience approach, targeting aging processes to prevent multimorbidity, via initiating broad clinical trials of potential geroprotective therapies, is recommended.

## Aging as a Risk Factor for Mortality in COVID-19 and Other Infections

The data on COVID-19 is clear on at least one point: Older adults are most vulnerable to hospitalization, disability and death following infection with the novel coronavirus [1]. As of April 2020, the Centers for Disease Control reported that 77% of all Covid-19 deaths in the US were people 65 and older. Among those infected, people 80 and older die at 200 times the rate of someone in their 20s (www.cdc.gov/nchs/nvss/vsrr/covid19/index.htm). Even prior to COVID-19, the risk of death attributed to pneumonia increased ~1,000 fold between ages 25 to 85 [2]. Therefore, a geroscience approach as described here is expected to be relevant when we tackle future infections, epidemics and pandemics.

## Geroscience

Understanding how drugs can delay aging and related diseases is part of a larger scientific endeavor supported by the National Institute on Aging and others called geroscience (www.nia.nih.gov/gsig). This approach aims to understand and ultimately modify the basic biology of aging and in so doing, develop new paradigms to treat multiple age-related chronic diseases at the same time. Geroscientists have long hypothesized that by targeting the biology of aging, all diseases of aging can be delayed [3]. Hallmarks of aging have been established and shown that they are all interconnected, thus targeting any single hallmark results in improvements in others [4,5]. In animal preclinical studies, health span and life span have been dramatically increased by targeting those hallmarks, using genetic tools and drugs, demonstrating that aging is a modifiable condition [6,7].

## Immune Dysfunction and Inflammaging are Hallmarks of Aging

Of particular importance are the hallmarks of immune dysfunction underlying the vulnerability of older adults to infections and the inflammation which accounts for the response to those infections [8]. But beyond improving immunity and inflammation to younger levels, such approaches also increase individuals’ resiliency to withstand the sickness itself, as the whole body needs to respond to this major stressor.

## What Do We Do Today?

Older people are at such risk in part because the vigor of our immune response flags as we age. In addition to age, many of us are also weakened by coexisting age-related conditions that diminish our resilience further. So, until a vaccine or treatment becomes available, how do we defend ourselves from infection beyond physical distancing and careful hand washing? We can focus on eating well, exercising as regularly as possible (at home and even in increasingly confined long-term care facilities), managing our stress, and getting enough sleep - behaviors we know help improve our immune responses at any age. Public health interventions that ensure access to healthy foods and safe environments are also critical.

## Geroprotectors-Gerotherapeutics

In addition to this healthy lifestyle, interventions with existing drugs with established safety profiles that target the biology of aging, immune mechanisms and resiliency (i.e. “geroprotectors” or “gerotherapeutics”), should be explored. While many geroprotectors have been successfully tested in pre-clinical settings, to date none of them has been approved as geroprotectors for use in humans. Consequently, self-medication with any of these compounds is highly discouraged.

One such drug is metformin which has been shown to target multiple hallmarks of aging [9] and increase health span and life span in animals. In addition, both clinical and observational trials in humans show that the use of this drug is associated with less type 2 diabetes mellitus (T2DM), cardiovascular diseases, cancer, cognitive decline and overall mortality [10]. Previous research from as far back as the 1940s, however, has pointed to metformin and metformin-like biguanide drugs as preventing influenza [11] and increasing in vivo and in vitro immune response in humans [12]. It is the first line of treatment for T2DM with 60 years of experience, exceptional safety record and is both generic and cheap.

Metformin has already indicated protective capacity against COVID-19. Thus, in a retrospective analysis of 283 T2DM patients from Wuhan, China, with confirmed COVID-19, investigators found no difference in the length of stay in hospital, but persons taking metformin had significantly lower in-hospital mortality (3 of 104, 2.9%) than those not taking metformin (22 of 179, 12.3%) [13]. Moreover, it was reported that diabetic women on metformin had ~20% decrease in mortality and ~80% decrease in the inflammatory marker TNFα [14].

A second line of drugs are mTOR inhibitors, which have been shown to increase healthspan and lifespan in almost all animals tested, from yeast to rodents. The mTOR inhibitor rapamycin reverses age-related declines in influenza vaccine response in mice [15] and two Phase 2 clinical trials completed by resTORbio Inc. showed that the rapamycin derivative everolimus could enhance influenza vaccine response in healthy elderly people [16,17]. The second Phase 2 trial also reported that those treated with mTOR inhibitors for 6 weeks had significantly fewer respiratory tract infections over the next year compared to those that received the placebo [17]. Thus, it appears that short-term inhibition of mTOR may confer protection not only against the flu, but also other common viruses including other coronaviruses.

## The Need to Advance Gerotherapeutics at This Time

Given the current public health crisis that is disproportionately affecting our aging population, it is imperative that we start discussing pragmatic approaches to rapidly implement the testing of such drugs in the face of the COVID-19 pandemic and an aging global population. At this stage, broad clinical trials of potential geroprotective therapies are needed, to enable extensive data collection and analysis of their potential benefits and indications.

Development and use of drugs like rapamycin and metformin by the at-risk population, notably older adults, may confer broad health benefits by targeting multiple aspects of biological aging and in this way raise the chances that these people can ward off the worst effects of COVID-19. Metformin for example is already used chronically by more than 100 million people around the world today. Metformin is broadly available and low-cost (just a little more than $.10/day). It should be tested rapidly to allow even a small percentage of older people to avoid hospitalization and death from COVID-19. The absolute benefits will be substantial. Rapamycin has also been used clinically for many years with a well-established dosing and safety profile. While significant side effects are associated with high-doses in patients undergoing daily rapamycin treatment, there is accumulating evidence that lower-dose treatment with rapamycin or its derivatives has minimal side-effects in healthy individuals [18,19] and may confer substantial improvements in immune function [16,17].

Randomized, controlled clinical trials to assess the ability of rapamycin, metformin and other potential geroprotective drugs [20], to boost response to an eventual COVID-19 vaccine in the elderly, as well as protect against COVID-19 infection altogether, could have a substantial impact on survival in vulnerable populations and should be pursued.

## Biotech and the Pipeline

Ongoing basic research on geroscience will produce a ‘pipeline’ into a whole raft of novel compounds that are being developed by biotech around the world. This may provide new insights into how we can modify the aging process and boost our immune response, perhaps even improving the performance of a vaccine for COVID-19 whenever it becomes available.

## A Geroscience Approach Would Benefit Not Only the Chronologically Old

Epidemiological data indicates that COVID-19 is particularly aggressive among older adults. However, even within that group there is a large heterogeneity of response, with some individuals suffering severe effects and/or death, while others recover with little more side effects than those observed in younger cohorts. In fact, heterogeneity of response is a major characteristic of older populations [21], which is why there is a need to identify those individuals, within an age cohort, that are physiologically younger or older than their chronological age. So, while a percentage of older adults are more robust than expected, so a fraction is more fragile. While no clear data exists yet in humans, those more fragile (i.e., physiologically older) are likely to be the ones who would most benefit from geroscience-based approaches to improve their health.

Just like there is heterogeneity among older adults, significant heterogeneity has been identified even among younger individuals [21]. It is likely that individuals who are chronologically young, but who display an advanced physiological age will also benefit from a geroscience approach. Those include, among others, high-risk individuals such as those with additional multimorbidity in addition to SARS-CoV-2 infection, as well as those with previous events that leave sequalae, such as controlled-HIV infection, previous chemotherapy or radiation, the disabled, obese and even poor people who cannot modify their interactions with the environment.

## Strategic Research Directions: Evaluation of Aging, Multimorbidity, and Frailty and the Effectiveness of Interventions Against Them

Multimorbidities in general and old-age multimorbidities in particular have proven to be the main risk factors for bad outcomes in COVD-19 patients [22,23]. Often, in older patients, multiple aging-related diseases are affected by multiple risk factors, further increasing the disability and mortality. Therefore, there is an urgent need to develop and implement analytical methodologies that would allow a diagnostic evaluation of the contribution of several co-existing diseases to COVID-19 outcomes. There is a need to identify either unique or combinatorial parameters to identify appropriate biomarkers, or risk factors for severity of disease in COVID-19 patients. The ability to weigh risks from multiple diseases and their combinations may also have critical implications for healthcare and social policy, both during the current crisis and in planning for future emergencies.

Particular contributing factors that may be crucial for outcomes in elderly subjects, for better or worse, may include their drug medications, e.g. ACE inhibitors [24], vaccinations, vitamins and other supplements, and other interventions more prevalent in and differentially affecting the older persons. The effectiveness of specific therapeutic regimens and protocols (such as different modes of oxygen delivery and resuscitation, drugs, vaccines and adjuvants) need to be evaluated and adjusted specifically for older patients whose management may dramatically differ from younger subjects with critical implications for outcomes.

The role of multimorbidities and interactions across diseases as synergistically affecting the outcomes should be addressed not only in retrospective analysis, but also when designing new experimental studies.

This multimorbidity evaluation should be combined with a systematic assessment of the aging and frailty status, using standardized methods and measures. Such standard methods and measures are needed to start creating prospective information on the differential vulnerability of older persons. Strategies must be developed for periodic frailty assessment with continuous longitudinal follow-up of older persons which may reveal not only global determinants that are conserved, but also local singularities involved in differential aging health around the world. Standard evaluation of biological and physiological age, with appropriate biomarkers, should be developed, both to assess and predict risks of aging-related ill health and to evaluate the effectiveness of potential geroscience therapies [25-27].

Wide public should be actively involved in such evaluation studies, not just as test subjects, but as active and empowered “citizen-scientists”. There is a need to increase public science education in the field of geroscience to inform the public and enhance their ability to evaluate evidence.

For the development and advantageous utilization of such evaluation methodologies, it is essential to improve access, openness, sharing and interoperability of clinical data on large cohorts of subjects, preferentially longitudinal data, including their clinical conditions and diseases (with or without COVID-19), demographic characteristics, and a wide set of evaluation parameters, including biochemical, metabolic, immunological, physiological, functional and other parameters, most of which are routinely available both in clinical practice and experimental settings. The development and utilization of such methods, on large clinical datasets, will help establish the most significant risk factors and their combinations, for multiple age-related diseases, and their joint contribution to COVID-19 outcomes, and facilitate recommendations for the effective combined therapy to mitigate COVID-19 and its risk factors.

## Policy Implications

The COVID-19 global emergency has emphasized to vast masses of people the vital need to prevent old-age multimorbidity, protect the elderly and improve their health span. Proponents of geroscience have argued for the importance of such preventive measures for many years. Now we see in front of our own eyes the disastrous consequences of the deficit in such preventive measures, and the portent this gap in our approach represents for the future. We are witnessing how this new infectious disease is wreaking havoc among individuals, the healthcare system and the entire social fabric around the world, while the rapid aging of the population represents the main risk factor and aggravating condition.

Therefore, arguably, one of the most important lessons to be learned from this pandemic, is the need to therapeutically address degenerative aging processes to prevent aging-related ill health as a whole. This understanding should translate to public health and research policies supportive of geroscience research, development and clinical application [5,28,29], improving the funding, incentives, education and institutional support for the field. With sufficient support and deployment, the preventive geroscience approach may help avoid or mitigate the effects of this and current devastating pandemics of aging-related ill health, presently and for the future.

Conquering the current pandemic will require a multipronged approach, including primarily an ‘offensive’ approach represented by the development of vaccines and treatments, as well as a ‘defensive’ approach focused on strengthening the resilience of affected individuals. Importantly, the offensive part of our arsenal requires the urgent development of a new vaccine, curative and palliative treatments for each successive pandemic and epidemic confronting the world. This aspect of our approach is unfortunately both slow and specific to the currently relevant virus or pathogen. In contrast, the defensive arm proposed here is pathogen-blind insofar as the interventions are pathogen independent. Therefore, a geroscience-focused response to the COVID-19 pandemic can be deployed not only against the current emergency, but the same approach will certainly be relevant to future infections, be them pandemic, epidemic, endemic, or even those affecting any one individual.
